# Intraoperative frozen section assessment of sentinel lymph nodes in the operative management of women with symptomatic breast cancer

**DOI:** 10.1186/1477-7819-6-69

**Published:** 2008-06-26

**Authors:** Rohanna Ali, Ann M Hanly, Peter Naughton, Constantino F Castineira, Rob Landers, Ronan A Cahill, R Gordon Watson

**Affiliations:** 1 Department of General Surgery, Waterford Regional Hospital, Waterford, Ireland; 2 Department of General Surgery, Our Lady's Hospital Cashel, Tipperary, Ireland; 3 Department of Histopathology, Waterford Regional Hospital, Waterford, Ireland

## Abstract

**Background:**

Maximisation of the potential of sentinel lymph node biopsy as a minimally invasive method of axillary staging requires sensitive intraoperative pathological analysis so that rates of re-operation for lymphatic metastases are minimised. The aim of this study was to describe the test parameters of the frozen section evaluation of sentinel node biopsy for breast cancer compared to the gold standard of standard permanent pathological evaluation at our institution.

**Methods:**

The accuracy of intraoperative frozen section (FS) of sentinel nodes was determined in 94 consecutive women undergoing surgery for clinically node negative, invasive breast cancer (37:T1 disease; 43:T2; 14:T3). Definitive evidence of lymphatic spread on FS indicated immediate level II axillary clearance while sentinel node "negativity" on intraoperative testing led to the operation being curtailed to allow formal H&E analysis of the remaining sentinel nodal tissue.

**Results:**

Intraoperative FS correctly predicted axillary involvement in 23/30 patients with lymphatic metastases (76% sensitivity rate) permitting definitive surgery to be completed at the index operation in 87 women (93%) overall. All SN found involved on FS were confirmed as harbouring tumour cells on subsequent formal specimen examination (100% specificity and positive predictive value) with 16 patients having additional non-sentinel nodes found also to contain tumour. Negative Predictive Values were highest in women with T1 tumours (97%) and lessened with more local advancement of disease (T2 rates: 86%; T3: 75%). Of those with falsely negative FS, three had only micrometastatic disease.

**Conclusion:**

Intraoperative FS reliably evaluates the status of the sentinel node allowing most women complete their surgery in a single stage. Thus SN can be offered with increased confidence to those less likely to have negative axillae hence expanding the population of potential beneficiaries.

## Background

Sentinel lymph node (SLN) biopsy is now established as an accurate, minimally invasive means of providing regional staging for primary breast cancer. As axillary clearance remains the standard of care for those with nodal spread[1], many centres however confine the use of SLN mapping to women with "*early*" or "*small*" breast cancer (i.e. T1 cancers in most instances)[2]. However, such a strategy deprives the benefits of minimally invasive lymphatic staging from those women who have disease that is locally advanced but still contained within the breast. Furthermore, means of preoperative T-staging are all too often inaccurate[3]. Any uncertainty surrounding the "completeness" of the initial surgery may weigh heavily on the minds of women undergoing lymphatic mapping due to the interval between operation and histological reporting.

An intraoperative means of cytopathological evaluation of sentinel nodes therefore has clear potential advantages although concerns over the potential for inconsistencies of conclusion between the rapid analysis and the formal paraffin section remain widespread. In this study, we describe our experience of routine intraoperative frozen section of sentinel nodes in women with invasive breast cancer but without overt lymphatic metastases in order to contribute to the emerging body of data regarding its practical reliability and clinical utility.

## Patients and Methods

The study protocol was reviewed fully and passed by the local hospital ethics committee.

### Patients and surgical technique

Consecutive, consenting, clinically node negative women with unifocal, invasive breast cancer undergoing surgical treatment for invasive breast cancer between December 2004 and December 2006 were studied. All patients underwent synchronous excision of the primary breast cancer (either by wide local excision or mastectomy) and sentinel node biopsy by one of two consultant surgeons (TC or RGW). All women were counselled and fully consented regarding the risk of requiring formal axillary clearance if their sentinel node was found to contain metastases either on frozen section examination or on subsequent conventional analysis. The degree of risk communicated was dependent mostly on the patient's tumour size (no patient had palpable lymphadenopathy). Under the study protocol, any evidence of nodal metastases in the sentinel node (either by frozen section or on subsequent formal pathological examination, including micrometastases (i.e. deposits <2 mm in diameter), were taken to mandate subsequent full axillary clearance at a second operation. This was decided, despite the recent WHO classification of isolated tumour cells as conferring a node-negative status, because of both the experimental nature of the technique in our hands and the ongoing controversy of whether such cells actually are prognostically important[4].

Our method of sentinel node identification incorporates the preoperative injection of both radioisotope and blue dye as previously described[5]. In brief, radioisotope tracer (99m-Tc-labelled colloidal rhenium sulphide-*Nanocis*, Cis Biointernational) was injected subcutaneously peri-tumourly one day prior to surgery. In cases of an impalpable tumour, fine wire localization (FWL) in combination with radio-isotope occult lesion localization (ROLL) methods was implemented[6]. Immediately preoperatively, after induction of general anesthesia, 3–5 mls of isosulphan blue dye (lymphazurin^®^, Ben Venue Laboratories Inc., Bedford OH) was also injected peritumorally. The SLN was then identified intra-operatively by visual inspection for blue dye in combination with searching for radiation counts with a hand held gamma-ray detection probe (Neoprobe^®^, Neoprobe Corporation). While the nodal tissue was being processed by frozen section (see below), the primary tumour was resected. The excised breast specimens were examined as usual to ascribe the type and grade (Bloom Richardson grading of hematoxylin and eosin-stained specimens) of the invasive component of the primary tumor, as well as its estrogen (ER) and progesterone receptor (PR) status.

### Pathological examination of the SLN

The same consultant pathologist (RL) examined all the specimens. Once localized and excised, the SLN(s) was sent immediately to the laboratory for frozen section examination. On average, this involved examination of three sections per node were (using OCT compound and freezing spray) unless obvious macroscopic metastases were apparent on the first slice examined. Patients found to have tumour cells in their SLN biopsy on frozen section analysis underwent immediate level II axillary lymph node dissection with all resected tissue being sent for standard pathological processing and review. Any remaining sentinel nodal tissue after frozen sectioning were fixed in formalin and embedded in paraffin with subsequent examination by H&E staining (again an average of 3 levels per node with a fixed distance between levels of 100 microns.) and, if any uncertainty pertained, immunohistochemistry. With regard to the nodes excised as part of an axillary clearance, smaller nodes were bisected and the whole node examined as per the sentinel node. Larger nodes were bisected and one half examined similarly. If no macroscopic metastases were evident, the other half was then also examined.

### Results and Statistical analysis

The results of the immediate, intraoperative FS assessment of the SLN were then compared with that of their delayed formal pathological examination with regard to nodal oncological status. ANOVA testing was used to examine for statistically significant differences between the groups with the Bonferroni correction used post hoc because of small numbers involved.

## Results

### Patient demographics and tumor characteristics

In total, 94 women were included in the study. Their mean age was 60 years (range 35–81, 73 being older than 50). Twenty eight patients had breast-conserving surgery (two required radiological guidance to localise the primary) while the remainder underwent mastectomy due either to high tumor:breast size, central location of the tumour or the patient's own preference. On final pathological analysis of the resected primary, 37 patients were found to have T1 tumours (2 were T1a, 9 were T1b and 26 were T1c) while 43 were T2 and 14 T3. Seventy six cancers were ductal adenocarcinoma while 18 were lobular in type. Nine were Grade I by Bloom Richardson scoring on H&E examintion while 50 were Grade II and 35 Grade III. Seventy eight of cancers were ER positive with 12 being both ER and PR positive.

### Sentinel Node Identification and frozen section analysis

There were no failed lymphatic mappings. The median number of SLN identified was 2 (mean 2.1 range 1–6). Intraoperative frozen section of the sentinel nodes identified tumour cells in 23 women with the subsequent formal H&E examination confirming the presence of lymphatic metastases in every one. Each of these patients underwent immediate level II axillary clearance on receipt of the frozen section report. The mean number of nodes subsequently harvested at axillary clearance was 15.3 (range 3–39). Sixteen patients had further nodal metastases identified in their non-sentinel nodes.

### Formal sentinel node analysis

Formal pathological examination identified nodal tumour deposits in seven patients whom the initial frozen section deemed not to have lymphatic dissemination. The frequency of false negative cases among those judged to be negative by frozen section analysis was therefore 9.9% while the false negative rate was 23%. Three of these patients actually had micrometastatic disease in their sentinel node (one of these patients required further immunohistochemical staining to confirm the presence of malignant cells on the formal specimen). All patients with involved nodes underwent level II axillary clearance at a second operation as per protocol. The mean number of nodes cleared at this operation was 11.5 (range 6–22). In two patients the sentinel node(s) were the only involved nodes while the other five women (included two of whom had only micrometastases evident in their sentinel node) had non-sentinel nodal metastases evident on full examination of the residual lymphatic basin.

### Comparison of frozen section analysis with formal pathological examination

The sensitivity and specificity rates as well as positive and negative predictive values both overall and by final pathological T stage are shown in Table 1. The accuracy of frozen section analysis of sentinel nodes according to tumour size was 97% for T1 tumours, 91% for T2 tumours and 86% for those with T3 disease. For those with Grade III cancers, it was 91%. The characteristics of those patients with false negative frozen sections in contrast to those with true negative and true positive frozen section results are shown in Table 2. As is evident from the table, patients with false negative sentinel node analysis by frozen section tended to have less sentinel nodes resected. They were also more likely to have larger tumours that were ER negative and were less likely to have Grade I tumours. On formal statistical analysis, however, no significant difference was demonstrable between the groups.

**Table 1:** Relationship between intraoperative frozen section (FS) of sentinel lymph nodes (SLN) and formal final histological analysis available after surgery.

Relationship between sentinel nodal analysis by Frozen Section and Conventional Histology				
		Paraffin section		
	
Crude data	Status	Positive	Negative	Total

Frozen Section	Positive	23	0	23
	Negative	7	64	71
	Total	30	64	94
				

Utility of intraoperative FS	Overall	T-stage		

*Parameter*	%	T1	T2	T3

Sensitivity	76	91%	69%	66%
Specificity	100%	100%	100%	100%
Positive Predictive Value	100%	100%	100%	100%
Negative predictive value	90%	97%	86%	75%

**Table 2:** Characteristics of patients by the frozen section status of the sentinel lymph nodes (SLN) found at the time of definitive surgery for the invasive primary.

SLN Category	Age	SLN No.	T size			Tumour Grade			ER and PR staining		
	
	Mean (yrs)	Mean	T1	T2	T3	I	II	III	ER positive	ER & PR positive	ER & PR negative
True Negative SLN by FS (*n *= 64)	61.5	2.02	34	24	6	8	33	22	53	39	8
True Positive SN by FS (*n *= 23)	62.8	2.1	10	9	4	0	10	13	21	19	2
Total True SN by FS (*n *= 87)	62.2	2.1	44	33	10	8	43	35	74	58	10
False Negative SN by FS (*n *= 7)	57.9	1.9	1	4	2	0	4	3	4	3	2

## Discussion

### The Role of SLN Biopsy

While there remains some dispute on the role of routine axillary clearance in selected patients with lymphatic metastases, most authorities recommend lymph basin dissection for *node positive *women in order to both fully debulk and stage the disease. Therefore, women found to have axillary disease after conventional pathological examination of their SLN require a second, separate operation to clear their (now scarred) axilla. A high likelihood of nodal disease undermines confidence in the potential advantages of SLN biopsy as any benefits to be derived from this technique are quickly outweighed by the physical risks and psychological detriment of a second reoperative procedure. Therefore many proponents of lymphatic mapping advocate that the technique be only implemented in patients with "early" breast cancer (that is, those with T1 cancers and a clinically negative axilla). Recent reports however have shown that the technique can function reliably in more advanced T-stages[7,8] although clearly such patients have a higher risk of nodal metastases and therefore a greater risk of needing a second operation if convention histological examination of the nodes is employed[9].

### Pitfalls of Preoperative Selection of Patients for SLN Biopsy

The ability to precisely measure tumours before their resection is however limited and clinical examination of the axilla is itself known to lack sufficient specificity and sensitivity to confidently guide appropriate patient selection for this technique[10]. How best preoperatively available measures of the primary tumor characteristics[11] can be used to select patients for SLN prior to definitive surgery is also unclear[12,13]. Furthermore, as selection criteria become increasingly stringent, a diminishing proportion of patients who could benefit from sentinel node biopsy are selected for the technique[5]. Additionally, it is uncertain whether recommendations based on T-stage alone can even be applied to symptomatic patients.

### Intraoperative SLN Analysis by Frozen Section Examination

An accurate means of intra-operative analysis of sentinel nodes has the potential to allow the completion of surgical treatment for a patient with breast cancer in a single session. While touch imprint cytology has been suggested as being a useful means of such analysis[14,15], currently, it is limited by its availability as well as variable sensitivity rates and, perhaps, most worryingly specificity rates that are less than 100% (therefore still risking a chance of condemning women without nodal disease to axillary clearance). Data on frozen section assessment however is emerging showing sufficient[16,17], and perhaps superior[18], intraoperative evaluation of nodal tumour burden, despite initial concerns that it consumes more of the specimen than cytological assessment. Analysis of published data to date shows that the accuracy of frozen section analysis with a combination of H&E staining and immunohistochemistry on sentinel lymph nodes lie between 73 to 96% [19-30].

### Discussion of Experience Presented in This Study

Overall the use of intraoperative frozen section analysis of sentinel node allowed 87/94 patients to have the axillary component of their surgery completed in a single step. Patients could also be informed of their status with this degree of confidence on awaking from anaesthesia. As has been suggested previously[24], the facility for intraoperative analysis of sentinel node status was particularly advantageous for those with larger tumors. Furthermore, the accurate, intraoperative selection of 23 of the 30 patients with axillary metastases allowed definitive axillary operation to be concluded at the index operation with confidence of therapeutic benefit. A second operation rate of 7% overall (23% in node positive patients) seems justifiable in the light of being able to maximize the numbers of node-negative women who can and should benefit from SLN. Rather than 45 T1 patients undergoing this procedure with five requiring a second operation for unexpected lymphatic disease, incorporation of frozen section analysis allowed all seventy one node-negative women in this series the benefits of minimally invasive determination of lymph node status at the expense of seven second procedures. The major undermining factor for frozen section analysis was in the detection of micrometastatic disease. While much attention is currently being focussed on micrometastatic detection in sentinel nodes, the clinical and prognostic significance of the finding of just a few malignant cells has not yet been fully elucidated.

## Conclusion

FS is again shown here to be a reliable method for evaluation of sentinel lymph nodes in patients with breast cancer. It allows immediate decision making regarding the need for ALND in the majority of patients.

## Competing interests

The authors declare that they have no competing interests.

## Authors' contributions

RA, AMH, PH, RL and CFC contributed to the study design and performance, data collection and analysis and manuscript preparation, RAC and RGW contributed to the study design, data analysis and presentation and manuscript preparation. All authors read and approved the final manuscript.
